# Modeling children’s weight growth trajectories: sex, country, and rural–urban differences in four low- and middle-income countries

**DOI:** 10.1186/s12887-025-06459-x

**Published:** 2025-12-31

**Authors:** Alemayehu Siffir Argawu, Begari Muniswamy, Begari Punyavathi

**Affiliations:** 1 Department of Statistics, Natural and Computational Sciences College, Ambo University, Ambo, Ethiopia; 2 Department of Statistics, College of Science and Technology, Andhra University, Visakhapatnam, 530003 India

**Keywords:** Child development, Weight growth trajectories, Logistic growth model, Country differences

## Abstract

**Background:**

Modeling children’s weight growth trajectories provides important insights into how biological and contextual factors shape development, yet most longitudinal research in low- and middle-income countries (LMICs) has focused on height rather than weight.

**Methods:**

We analyzed data from the Younger Cohort of the Young Lives study in Ethiopia, India, Peru, and Vietnam (2002–2016). Children with fewer than five weight measurements or missing key variables were excluded. The final analytic sample included 7,140 children contributing 35,700 observations. Weight trajectories from ages 1 to 15 years were modeled using a three-parameter logistic nonlinear mixed-effects model with fixed effects for sex, country, and rural–urban residence, and random effects on all three parameters to capture between-child heterogeneity.

**Results:**

The model revealed marked variability in asymptotic weight, timing, and growth rate. Peak growth velocity occurred at 13.9 years (~ 3.3 kg/year), with males peaking earlier than females and urban children peaking earlier than rural peers. Across countries, children in Peru and Vietnam had higher growth velocities and asymptotic weights than those in Ethiopia and India. Urban children consistently showed higher trajectories than rural children. Variance components and intraclass correlations confirmed substantial between-child heterogeneity in growth patterns.

**Conclusions:**

Children’s weight trajectories vary significantly by sex, country, and rural–urban residence. The findings highlight persistent disadvantages in rural Ethiopia and India alongside emerging risks of overweight in more urbanized Peru and Vietnam. Policies tailored to local contexts are needed to promote healthy weight growth across childhood and adolescence.

**Supplementary Information:**

The online version contains supplementary material available at 10.1186/s12887-025-06459-x.

## Background

 Modeling children’s weight growth trajectories provides critical insights into nutritional status, health, and development across the life course. While most longitudinal studies in low- and middle-income countries (LMICs) have concentrated on height, far fewer have examined weight, despite its sensitivity to both biological processes and environmental influences [1–3]. Weight trajectories can reveal early risks of undernutrition and later risks of overweight, thereby informing interventions that support healthy growth [4, 5].

Evidence from LMICs shows that rapid economic, social, and dietary transitions are reshaping child growth patterns. Persistent undernutrition continues to affect many rural and disadvantaged populations, particularly in sub-Saharan Africa and South Asia, while rising overweight and obesity are increasingly observed in urban settings of Latin America and Asia [6–8]. These dual burdens highlight the importance of documenting and comparing weight growth trajectories across diverse country contexts.

Most prior longitudinal analyses have emphasized child height and stunting as markers of nutritional status [9–11]. While these remain important, weight trajectories may better capture short-term responses to nutritional intake, infections, and lifestyle changes [12]. Research in Ethiopia, India, Peru, and Vietnam—the Young Lives countries—has demonstrated substantial heterogeneity in children’s growth experiences, yet comprehensive modeling of weight trajectories over childhood and adolescence remains limited [13, 14].

The present study applies a logistic nonlinear mixed-effects model to data from the Younger Cohort of the Young Lives study in Ethiopia, India, Peru, and Vietnam. Our primary aim was to model children’s weight growth trajectories from ages 1 to 15 years, while examining differences by sex, country, and rural–urban residence. By characterizing these patterns and their variability, we provide evidence to inform policies that address both persistent undernutrition and emerging risks of overweight in diverse LMIC settings [15–17].

## Methods

### Study design and data source

The Young Lives (YL) study is a longitudinal investigation of childhood poverty conducted in Ethiopia, India (Andhra Pradesh & Telangana), Peru, and Vietnam [17]. It follows two cohorts of children recruited in 2002: a Younger Cohort of ~ 2,000 children per country (aged 6–18 months, ≈ 1 year on average at baseline) and an Older Cohort of ~ 1,000 children aged 7.5–8.5 years. The present analysis uses only the Younger Cohort, with weight measured across five rounds from 2002 to 2016 (ages 1–15 years) [15, 16]. Children were recruited from 20 sentinel sites in each country, which were purposively selected to capture geographic and socioeconomic diversity, with oversampling of poorer households; within sites, households were randomly sampled [15, 18]. Attrition in the Younger Cohort remained low (2–8% by Round 5) [19]. Key design and data quality details are provided in Supplementary Table 1.

### Sampling, recruitment, and ethics

Recruitment was conducted at the household level, with eligibility defined by child age (6–18 months at Round 1) and residence in a sentinel site. Caregiver informed consent was obtained at baseline, and child assent was added in later rounds. Ethical approval was granted by national committees in each country and by the University of Oxford [18, 20]. To assess potential selection bias, we compared baseline characteristics of children included in the analytic sample with those excluded due to < 5 weight measures or missing key covariates (Results, Table 2).

### Inclusion, exclusion, and sample size

**Fig. 1:**
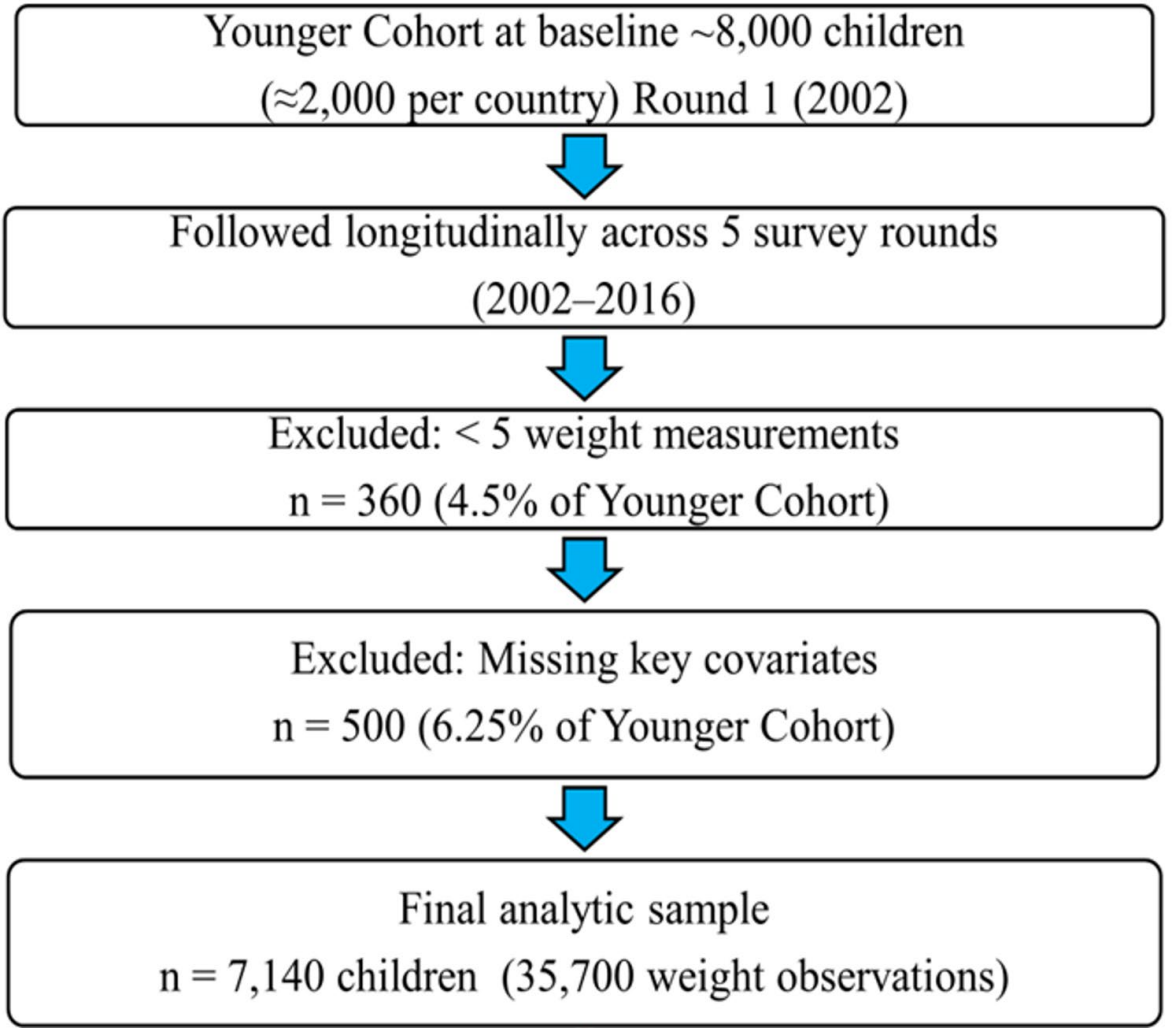
Participant flowchart and analytic sample

The Younger Cohort originally included ~ 8,000 children across the four countries. For this analysis, we excluded 360 children (4.5%) with fewer than five weight measures and 500 (6.25%) with missing values on key variables (sex, country, or residence). These exclusions were mutually exclusive, yielding a final analytic sample of 7,140 children who contributed 35,700 weight observations. To illustrate the selection process, Fig. 1 presents the sample flow, while Table 1 summarizes overall cohort characteristics and Table 2 compares baseline characteristics of included versus excluded children.

**Table 1:** Baseline characteristics of the analytic sample (*n* = 7,140). Distribution by country, sex, and residence, with total number of weight observations (*n* = 35,700)

Characteristic	Number of Children	Number of Observations	%
Country			
India	1,862	9,310	26.1
Ethiopia	1,665	8,325	23.3
Peru	1,761	8,805	24.7
Vietnam	1,852	9,260	25.9
Sex			
Male	3,728	18,640	52.2
Female	3,412	17,060	47.8
Residence			
Urban	4,426	22,129	62.0
Rural	2,714	13,571	38.0
Total	7,140	35,700	100

**Table 2:** Comparison of included versus excluded children at baseline. Characteristics include sex, residence, and mean baseline weight (kg ± SD)

Characteristic	Included (*n* = 7,140)	Excluded (*n* = 860)	*p*-value*
Sex, % female	47.8%	49% (approx.)	0.62
Residence, % rural	38.0%	40% (approx.)	0.55
Mean baseline weight (kg ± SD)	8.4 ± 1.2	8.3 ± 1.3	0.27

*P-values from χ² tests for categorical variables and t-test for continuous variables

### Measures

Weight (kg) was measured at each round by trained fieldworkers using standardized protocols and calibrated digital scales. To ensure accuracy, Young Lives implemented standardized measurer training, regular equipment calibration, and close field supervision. In addition, approximately 10% of children were re-measured (duplicate weight checks) at each round to assess reliability, consistent with Young Lives fieldwork protocols [21]. Covariates included sex (male/female), country (Ethiopia, India, Peru, Vietnam), and place of residence (rural/urban), recorded at the time of measurement. Coding was harmonized across rounds for consistency.

### Statistical analysis

We modeled individual growth trajectories using a three-parameter logistic curve within a nonlinear mixed-effects framework [22, 23]. The logistic function was specified as:

$$\:f\left(\mathrm{a}\mathrm{g}\mathrm{e}\right)=\frac{\mathrm{A}\mathrm{s}\mathrm{y}\mathrm{m}}{1+\mathrm{e}\mathrm{x}\mathrm{p}\left[-\frac{\left(\mathrm{a}\mathrm{g}\mathrm{e}-\mathrm{X}\mathrm{m}\mathrm{i}\mathrm{d}\right)}{\mathrm{S}\mathrm{c}\mathrm{a}\mathrm{l}}\right]}\:,$$

where Asym represents the maximum expected weight, Xmid the age at maximum growth rate, and Scal the steepness of the curve. Random effects were included on all three parameters (Asym, Xmid, Scal) to account for between-child heterogeneity, while fixed effects for sex, country, and residence were estimated to assess systematic group differences. For clarity, all symbols are defined in Supplementary Table 2.

Model estimation was performed using the nlme package in R (version 4.4.1) [24, 25]. Competing models were compared using AIC and BIC criteria, likelihood ratio tests, and residual diagnostics. Biological plausibility of parameter estimates was also considered. Robustness was evaluated through sensitivity analyses excluding outliers and by fitting models with log-transformed weight values.

## Results

### Participant flow and analytic sample

From the Younger Cohort (~ 8,000 children at baseline), 7,140 children met inclusion criteria, contributing 35,700 weight observations across five rounds (2002–2016) (Fig. 1). Baseline characteristics of the analytic sample are shown in Table 1. A comparison of included versus excluded children at baseline is presented in Table 2.

### Exploratory summaries

**Fig. 2:**
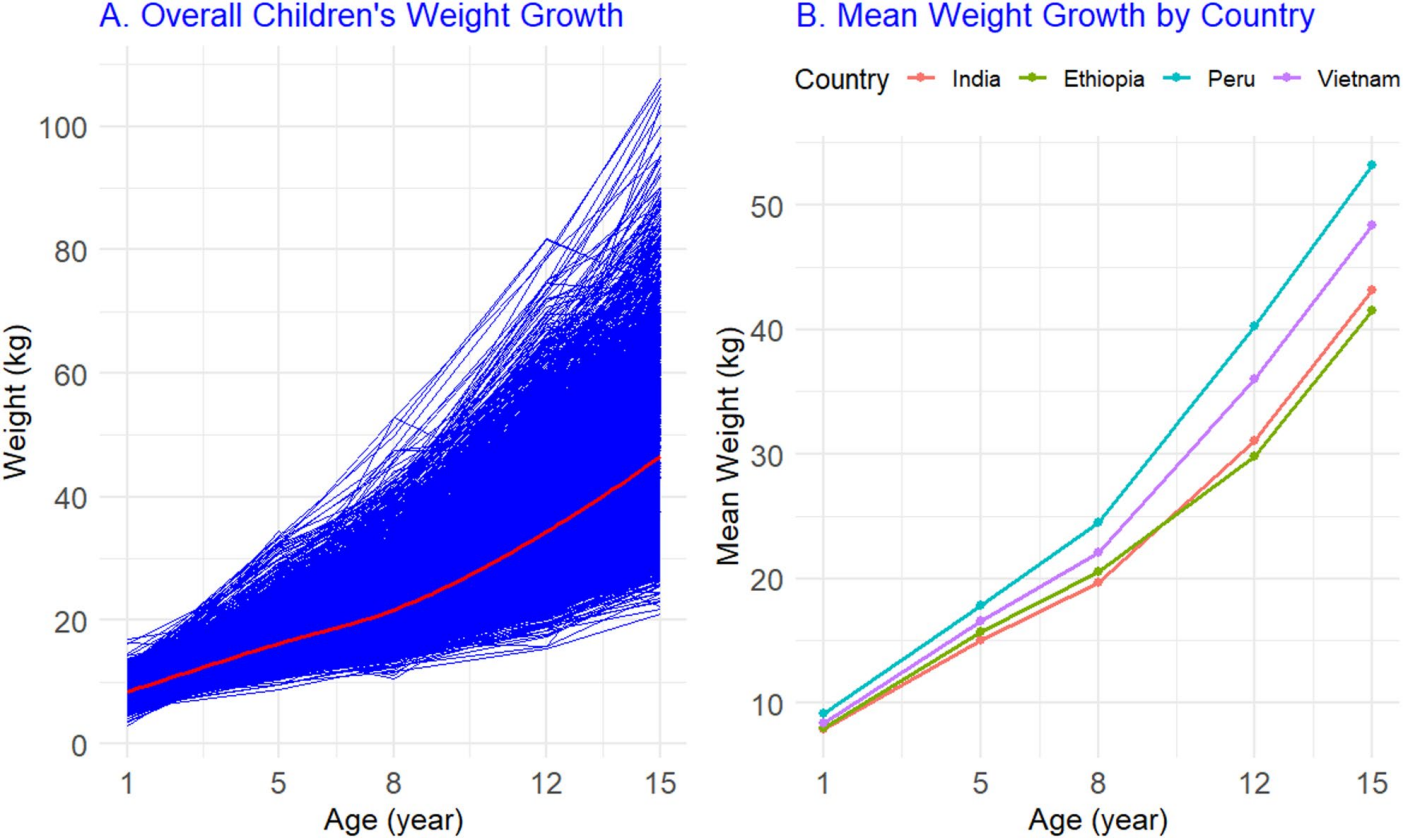
Overall smoothed weight growth trajectories from ages 1 to 15 years **A** and raw mean weights by country across five survey rounds from 2002 to 2016 **B**

Descriptive analyses revealed nonlinear increases in mean weight between ages 1 and 15 years, with marked variability by country, sex, and residence. Figure 2A shows the overall smoothed trajectories of children’s weight growth from ages 1 to 15, while Fig. 2B shows raw mean weights by country across the five survey rounds. Peru consistently showed the highest means, followed by Vietnam, India, and Ethiopia. These descriptive findings are supported by Table 3, which reports mean weights by round, country, sex, and residence.

**Table 3:** Mean weight (kg) by survey round, country, sex, and residence

Subgroup	Round 1 (2002)	Round 2 (2006)	Round 3 (2009)	Round 4 (2013)	Round 5 (2016)
By Country					
Ethiopia	7.7 ± 1.1	13.4 ± 2.3	21.5 ± 3.8	35.2 ± 6.4	44.9 ± 8.2
India	8.3 ± 1.0	14.6 ± 2.6	23.7 ± 4.0	39.0 ± 6.8	50.8 ± 9.5
Peru	9.2 ± 1.2	15.8 ± 2.7	26.2 ± 4.3	43.2 ± 7.0	57.6 ± 10.3
Vietnam	8.5 ± 1.1	15.1 ± 2.5	24.9 ± 4.1	41.5 ± 6.9	55.1 ± 9.8
By Sex					
Male	8.4 ± 1.2	15.0 ± 2.6	25.0 ± 4.2	42.0 ± 7.1	54.9 ± 9.9
Female	8.3 ± 1.1	14.7 ± 2.5	24.2 ± 4.0	40.6 ± 6.8	53.2 ± 9.7
By Residence					
Urban	8.6 ± 1.2	15.2 ± 2.6	25.1 ± 4.1	42.3 ± 7.0	55.3 ± 9.9
Rural	8.1 ± 1.1	14.5 ± 2.5	24.0 ± 4.0	40.1 ± 6.7	52.8 ± 9.6

**Fig. 3:**
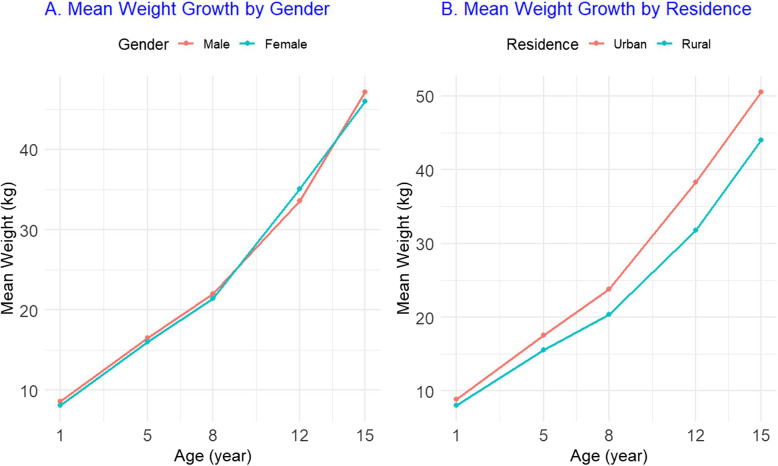
Mean weight growth trajectories by sex **A** and residence **B**

Figure 3A shows mean trajectories by sex, while Fig. 3B shows trajectories by residence. Male children exhibited slightly higher gains during adolescence than females. Urban children were heavier in earlier rounds, whereas rural children showed later peak ages and longer growth periods. These patterns are also reported in Table 3.

### Choice of outcome transformation

We compared three logistic model specifications: raw weight, cleaned weight, and log-transformed weight. The log-transformed outcome provided the best statistical fit (lowest AIC/BIC) and produced homoscedastic residuals. Therefore, the log-transformed weight was selected for subsequent nonlinear mixed-effects modeling (Table 4).

**Table 4:** Model fit indices for outcome transformation

Outcome transf.	AIC	BIC	Log-likelihood	Residual pattern	Decision
Raw weight	286,501	286,535	–117,233	Heteroscedastic	Rejected
Cleaned weight	279,986	280,020	–111,531	Slight skew	Rejected
Log-transf. weight	26,965	26,999	7,466	Approx. normal	**Selected**

### Nonlinear model family comparison

Several nonlinear families were considered, including logistic, Gompertz, von Bertalanffy, and Brody. Although the Gompertz model achieved a substantially lower AIC (indicating superior statistical fit), it implied biologically implausible asymptotic weights (> 125 kg). The logistic model, with its realistic asymptote (≈ 87 kg), best balanced statistical performance and biological plausibility on the back-transformed weight and was therefore selected for all subsequent analyses (Table 5).

**Table 5:** Nonlinear family comparison

Model family	AIC	BIC	Biological plausibility	Decision
Logistic	−14,923	−14,889	Asymptote ≈ 87 kg	Selected
Gompertz	−15,239	−15,205	Asymptote > 125 kg	Implausible
von Bertalanffy	−15,610	−15,576	Poor fit at younger ages	Rejected
Brody	−15,523	−15,489	Implausible growth rates	Rejected

### Model’s fixed effects estimates

The selected model was a three-parameter logistic nonlinear mixed-effects model fit to log-transformed weight. The model included fixed effects for sex (reference = Male), country (reference = India) and residence (reference = Urban), and random effects on all three parameters (Asym, Xmid, Scal) to capture between-child heterogeneity. Estimation was performed in R (nlme); diagnostics and sensitivity checks are reported in the Supplementary Table 3.

Table 6 presents fixed-effect estimates (back-transformed to kg). Key population-level findings were: females had lower asymptotic weights than males (Asym_(female): log-estimate = − 0.06; back-transformed ≈ 82.3 kg, 95% CI: 81.3–83.3, *p* < 0.001). Country differences (vs. India) were large: Ethiopia had a substantially lower asymptote (Asym_(Ethiopia): log-estimate = − 0.33; back-transformed ≈ 65.4 kg, 95% CI: 62.9–67.9, *p* < 0.001), Peru had a substantially higher asymptote (Asym_(Peru): log-estimate = 0.12; back-transformed ≈ 98.5 kg, 95% CI: 93.9–103.3, *p* < 0.001), while Vietnam did not differ significantly from India (Asym_(Vietnam): log-estimate ≈ − 0.001; back-transformed ≈ 87.3 kg, 95% CI: 84.0–90.6, *p* = 0.963). Residence differences were small and not statistically significant (Asym_(Rural): log-estimate = 0.01; back-transformed ≈ 88.2 kg, 95% CI: 85.9–90.5, *p* = 0.466), indicating that adjusted asymptotic weights were similar for urban and rural children.

**Table 6:** Fixed effects estimates for asymptotes (back-transformed to kg). Population-level effects for sex, country, and residence, with 95% confidence intervals and p-values

Predictor	Estimate (log scale)	SE	t-value	*p*-value	Back-transf. estimate (kg)	95% CI (kg)
Sex						
Female vs. Male	−0.06	0.01	−9.52	< 0.001	82.3	81.3–83.3
Country						
Ethiopia vs. India	−0.33	0.02	−17.42	< 0.001	65.4	62.9–67.9
Peru vs. India	+ 0.12	0.02	6.37	< 0.001	98.5	93.9–103.3
Vietnam vs. India	−0.001	0.02	−0.05	0.963	87.3	84.0–90.6
Residence						
Rural vs. Urban	+ 0.01	0.01	0.73	0.466	88.2	85.9–90.5

### Variance components and clustering

Table 7 shows that a large proportion of variance in the asymptotic weight (Asym) was attributable to between-child differences (ICC = 0.72 on the log scale; 0.69 after back-transformation), while residual within-child variance was modest (ICC ≈ 0.11–0.12). Similarly, high ICCs for the midpoint (Xmid = 0.83) and scale (Scal = 0.92) parameters confirm substantial heterogeneity in timing and growth rates across children. These results support inclusion of random effects on all three parameters in the logistic nonlinear mixed-effects model.

**Table 7:** Variance components, correlations, and intraclass correlations (ICCs) from the three-parameter logistic nonlinear mixed-effects model

Parameter	SD (log-weight scale)	SD (back-transf.)	Correlation coefficients	ICC (log-weight)	ICC (back-transf.)
Asym ($\:{\boldsymbol{\varphi\:}}_{1}$)	0.213	0.046	–	0.72	0.69
Xmid ($\:{\boldsymbol{\varphi\:}}_{2}$)	0.681	–	$\:{r}_{{\varphi\:}_{1}{\varphi\:}_{2}}=0.46$	0.83	–
Scal ($\:{\boldsymbol{\varphi\:}}_{3}$)	1.063	–	$\:{r}_{{\varphi\:}_{1}{\varphi\:}_{3}}=0.52,\:{r}_{{\varphi\:}_{2}{\varphi\:}_{3}}=0.34$	0.92	–
Residual	0.098	0.0096	–	0.11	0.12

### Peak growth velocities

**Fig. 4:**
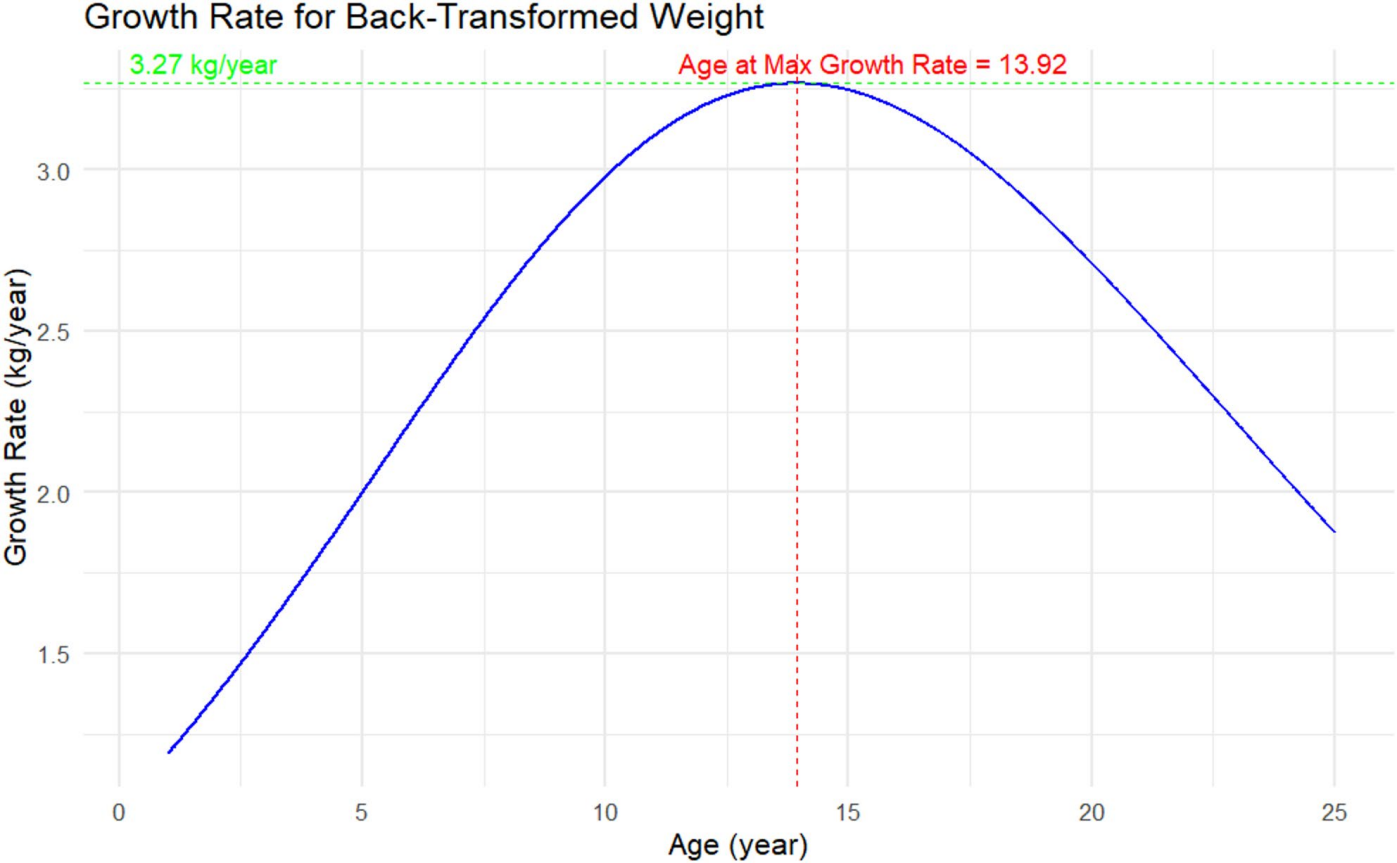
Overall peak weight growth velocity curve. Estimated instantaneous growth velocities (kg/year) derived from logistic nonlinear mixed-effects model

**Fig. 5:**
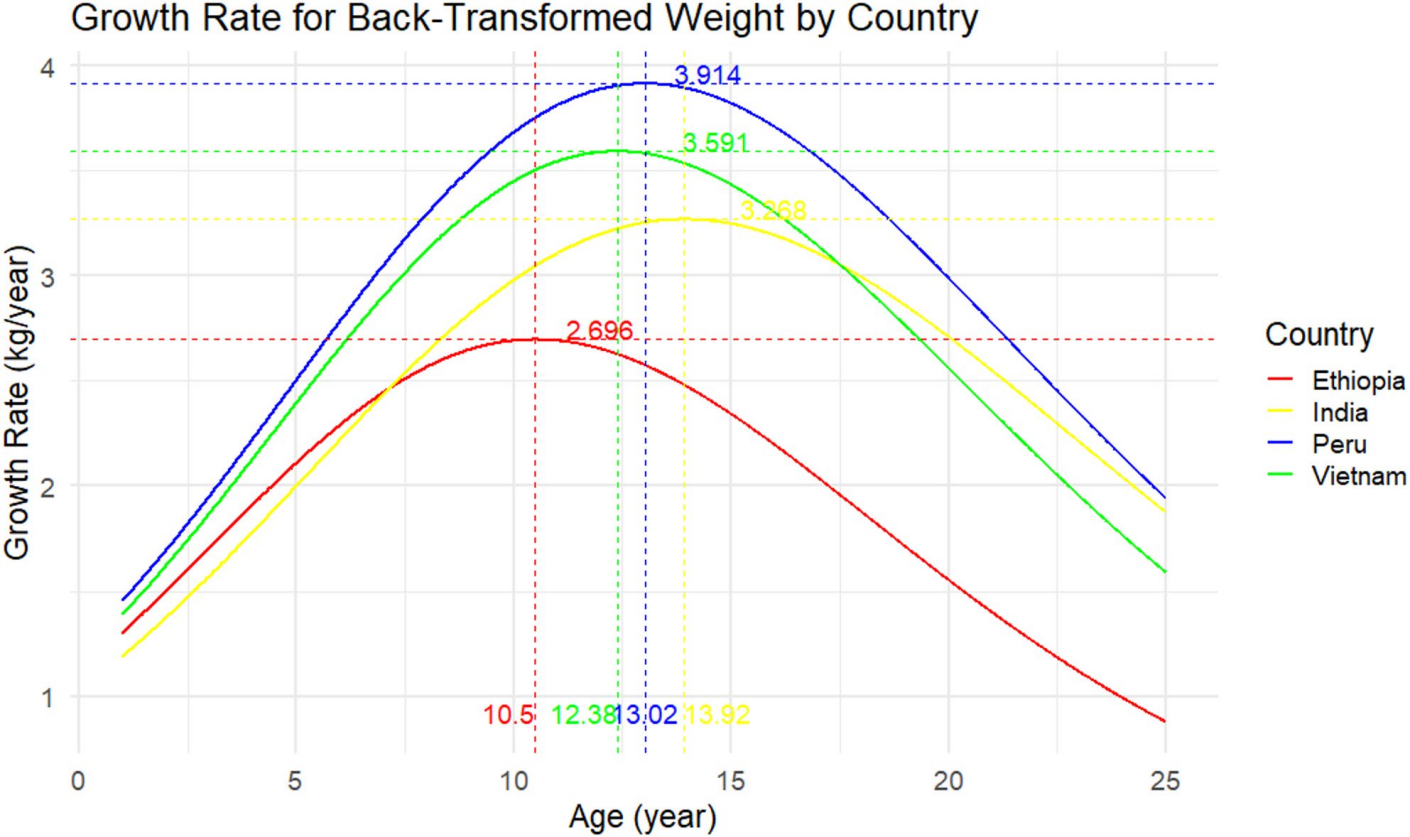
Peak weight growth velocity curves by country

**Fig. 6:**
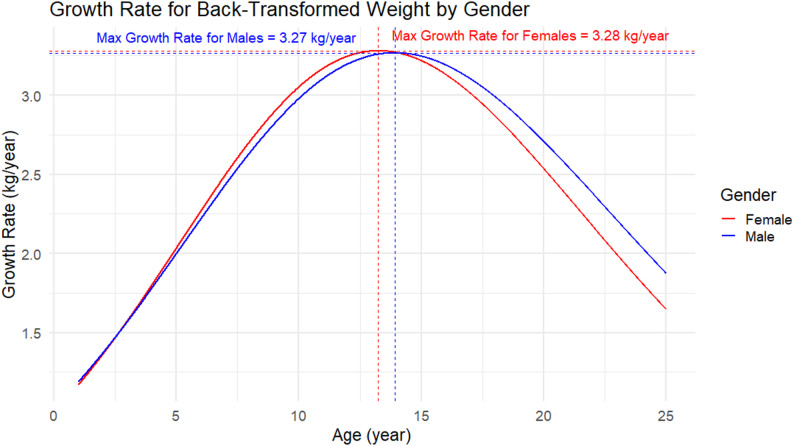
Peak weight growth velocity curves by sex

**Fig. 7:**
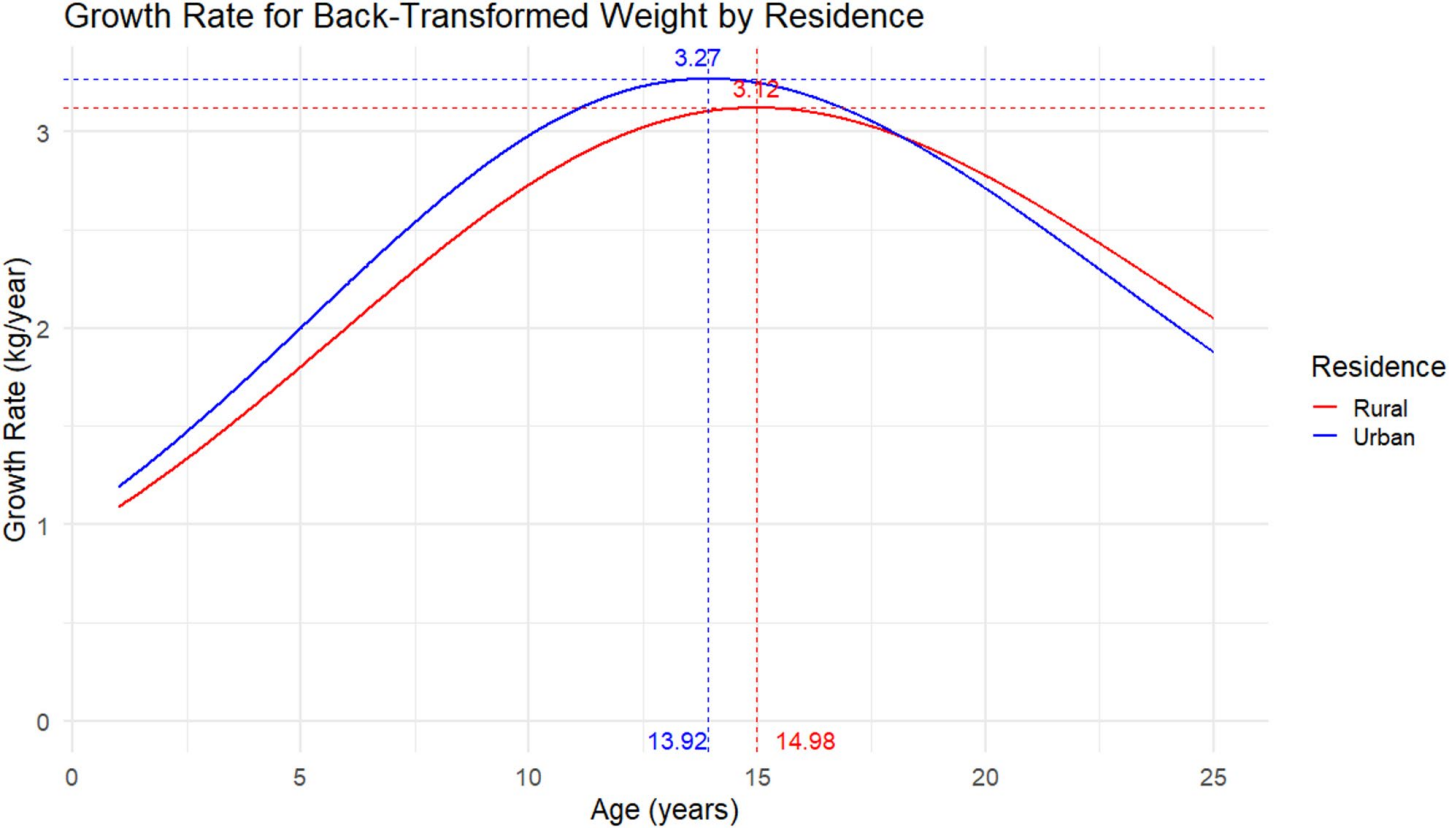
Peak weight growth velocity curves by residence

Empirical estimates from the fitted curves indicated that the overall peak weight velocity was approximately 3.27 kg/year, occurring at 13.9 years of age (Fig. 4). Country-specific patterns showed notable variation: children in Ethiopia peaked earlier, at around 10.5 years with a maximum velocity of 2.7 kg/year, whereas those in India peaked later at 13.9 years with 3.3 kg/year. Children in Peru experienced the highest peak velocity, reaching 3.9 kg/year at 13.0 years, while Vietnamese children peaked at 3.6 kg/year at 12.4 years. By sex, the curves indicated that males reached their peak at 13.9 years (3.27 kg/year), slightly earlier than females, who peaked at 14.3 years (3.28 kg/year). Differences by residence were also observed: urban children peaked at 13.9 years with a maximum of 3.27 kg/year, whereas rural children exhibited a slower and later peak velocity of 3.12 kg/year at 15.0 years. These subgroup-specific velocity patterns are illustrated in Figs. 5 and 6 (country, sex and residence curves) Figure 7.

### Model diagnostics

**Fig. 8:**
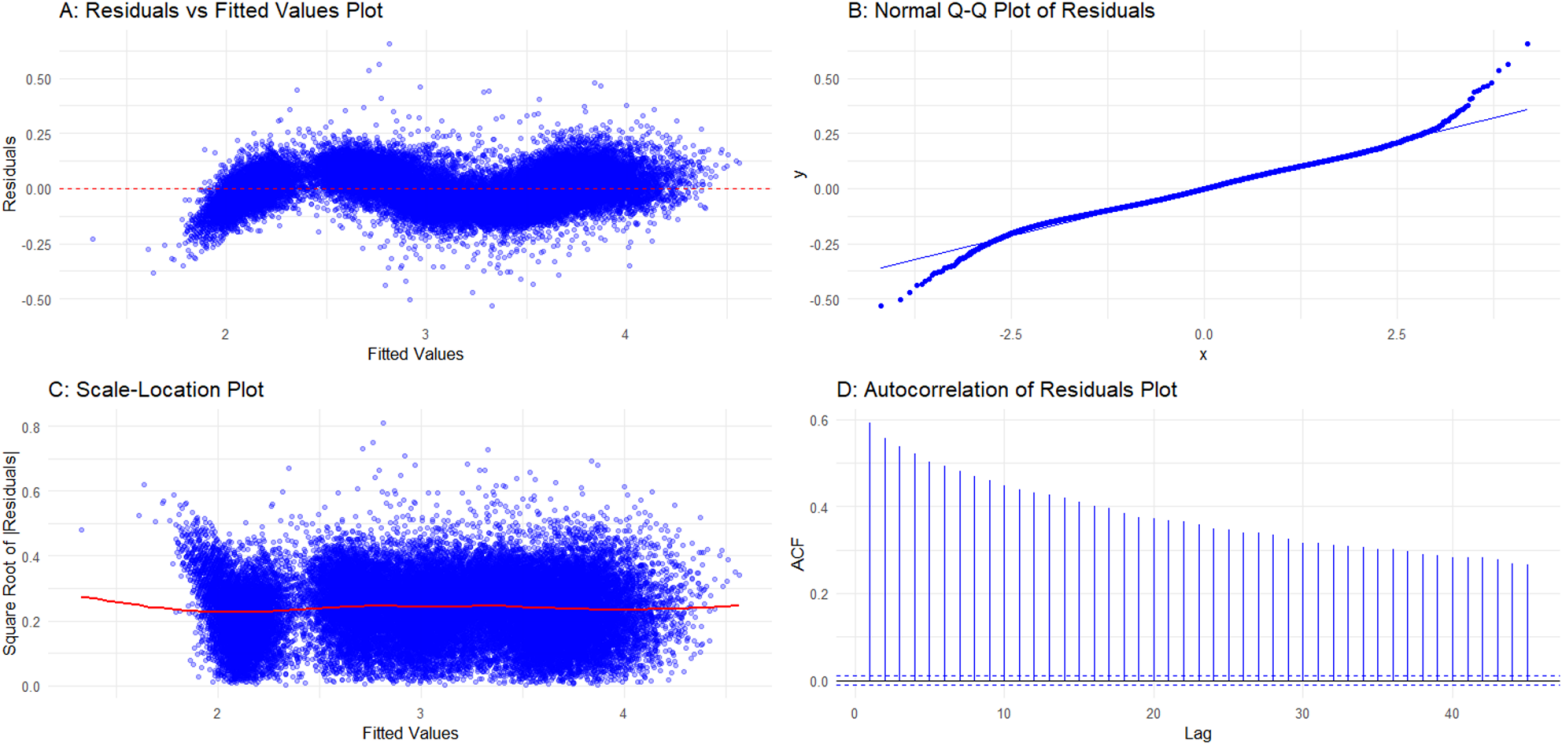
Model diagnostics by residuals versus fitted values **A** normal Q–Q plot **B** scale-location plot **C** and autocorrelation function (ACF) plot of residuals **D**

Diagnostic plots (Fig. 8) shows that residuals were approximately normally distributed with no major heteroscedasticity or autocorrelation. Sensitivity analyses (excluding outliers, testing alternative models) produced consistent results (Supplementary Table 3).

### Sensitivity analyses

Results were robust to alternative model specifications (Supplementary Table 3). Excluding statistical outliers (*n* ≈ 200 children) produced nearly identical parameter estimates. Removing the random effect on the midpoint parameter (Xmid) yielded slightly shifted estimates and poorer overall model fit (higher AIC/BIC). Alternative nonlinear families, such as the Gompertz model, resulted in implausible asymptotic weights (> 125 kg). These findings support the choice of the three-parameter logistic nonlinear mixed-effects model as both statistically adequate and biologically plausible.

## Discussion

In this study, we applied a three-parameter logistic nonlinear mixed-effects model to characterize children’s weight growth trajectories from early childhood to mid-adolescence across four low- and middle-income countries (Ethiopia, India, Peru, and Vietnam). The model captured both the overall growth patterns and the substantial between-child heterogeneity in asymptotic weight, timing, and growth rates. Importantly, we identified consistent differences by sex, country, and rural–urban residence, suggesting that both biological processes (e.g., pubertal timing, body composition) and contextual determinants (nutrition, socioeconomic conditions, and cultural practices) influence weight development. These findings extend prior longitudinal analyses that have predominantly focused on height trajectories rather than weight [1, 5, 26], and add to emerging evidence from Ethiopia and other LMICs that highlights the value of modeling weight growth trajectories [12, 13].

We found that children from urban areas, and those in Peru and Vietnam, had higher growth rates and attained greater asymptotic weights compared with rural children and those in Ethiopia or India. These disparities are consistent with known differences in socioeconomic development, dietary diversity, and access to health services across settings [4, 9]. Urban residence is often associated with greater food security and healthcare availability, which can support linear and ponderal growth, though it may also increase risks of overweight in later adolescence [27]. By contrast, rural children, particularly in Ethiopia and India, face higher burdens of undernutrition and infectious disease, which constrain weight gain trajectories [28]. Evidence from LMICs, including Ethiopia, confirms persistent rural disadvantages in child growth [12, 13, 15], underscoring how structural inequalities translate into divergent weight patterns during childhood and adolescence.

We also observed sex differences in weight growth trajectories, with males reaching peak velocities earlier and at slightly higher magnitudes than females. This pattern is consistent with known biological differences in body composition and pubertal development, where males typically experience more rapid gains in lean mass during adolescence [29, 30]. However, sex differences in growth are not purely biological. In many low- and middle-income settings, sexed caregiving practices and cultural preferences can influence dietary allocation, healthcare access, and exposure to physical activity [31, 32]. Prior evidence from LMICs, including Ethiopia, suggests differential feeding and morbidity patterns by sex, which may partly explain observed differences [12, 13]. Thus, the trajectories we observed likely reflect an interplay of physiological and social determinants.

### Strengths and limitations

This study has several methodological strengths. We used large, longitudinal cohorts followed over 15 years across four diverse LMICs, enabling robust modeling of weight trajectories from early childhood into adolescence. The application of a logistic nonlinear mixed-effects model allowed us to account for both fixed effects of key covariates and random effects that captured between-child heterogeneity. These features strengthen the reliability and generalizability of our findings. Nevertheless, some limitations must be acknowledged. First, attrition occurred across survey rounds, and although retention in Young Lives was relatively high compared with other long-term cohort studies [21], differential loss to follow-up may have introduced bias. Second, children with fewer than five weight measures were excluded to ensure robust trajectory estimation, which may limit representativeness of the most disadvantaged subgroups. Third, weight was measured without concurrent data on body composition or pubertal staging, preventing us from distinguishing lean mass from fat mass changes during adolescence. Finally, while we adjusted for sex, country, and rural–urban residence, other unmeasured contextual factors (e.g., household diet, parental education, local health services) likely contribute to observed differences and warrant further study.

### Public health and Policy implications

The findings have important public health and policy implications. Weight growth trajectories provide a sensitive indicator of how structural and household-level determinants shape child development. The observed country and rural–urban differences highlight the need for targeted interventions to address undernutrition in disadvantaged populations, particularly in rural Ethiopia and India, while also monitoring the emerging risk of overweight and obesity in more urbanized settings such as Peru and Vietnam [9, 27]. Interventions should integrate nutrition programs, maternal and child health services, and adolescent health initiatives, tailored to the local context. From a research perspective, future work should incorporate dietary intake, body composition, and pubertal development data to better elucidate the mechanisms underlying observed growth patterns. Longitudinal modeling approaches such as those applied here are valuable tools to inform policies that promote healthy growth trajectories across the life course.

## Conclusion

Children’s weight growth trajectories from early childhood to adolescence varied substantially by sex, country, and rural–urban residence across Ethiopia, India, Peru, and Vietnam. Using a logistic nonlinear mixed-effects model, we showed marked heterogeneity in asymptotic weight, timing, and growth rates, highlighting both biological and contextual influences. These findings underscore the need for context-specific nutrition and health policies that address persistent rural disadvantages while also responding to emerging risks of overweight in more urbanized settings.

## Supplementary Information

Supplementary material 1.

Supplementary material 2.

Supplementary material 3.

## Data Availability

The datasets for this study are publicly available from the UK Data Service upon registration and request (https://beta.ukdataservice.ac.uk/datacatalogue/series/series? id=2000060).

## Abbreviations

LMIC: Low- and middle-income country
YL: Young Lives study
ICC: Intra-class correlation
SD: Standard deviation
WHO: World health organization

## References

[1] WHO Multicentre Growth Reference Study Group, de Onis M. WHO child growth standards based on length/height, weight and age. Acta Paediatr. 2006;95:76–85. 10.1111/j.1651-2227.2006.tb02378.x 16817681

[2] Cole TJ, Freeman JV, Preece MA. British 1990 growth reference centiles for weight, height, body mass index and head circumference fitted by maximum penalized likelihood. Stat Med. 1998;17:407–29. 9496720

[3] Marceau K, Ram N, Houts RM, Grimm KJ, Susman EJ. Individual differences in boys’ and girls’ timing and tempo of puberty: modeling development with nonlinear growth models. Dev Psychol. 2011;47(5):1389–404. 21639623 10.1037/a0023838 PMC3928626

[4] Black RE, Victora CG, Walker SP, Bhutta ZA, Christian P, De Onis M, et al. Maternal and child undernutrition and overweight in low-income and middle-income countries. Lancet. 2013;382(9890):427–51. 23746772 10.1016/S0140-6736(13)60937-X

[5] Adair LS, Fall CH, Osmond C, Stein AD, Martorell R, Ramirez-Zea M, et al. Associations of linear growth and relative weight gain during early life with adult health and human capital in countries of low and middle income: findings from five birth cohort studies. Lancet. 2013;382(9891):525–34. 23541370 10.1016/S0140-6736(13)60103-8 PMC3744751

[6] Popkin BM. Nutrition transition and the global diabetes epidemic. Curr Diab Rep. 2015;15(9):64. 26209940 10.1007/s11892-015-0631-4 PMC4942180

[7] Alem AZ, Yeshaw Y, Liyew AM, Tessema ZT, Worku MG, Tesema GA, et al. Double burden of malnutrition and its associated factors among women in low and middle income countries: findings from 52 nationally representative data. BMC Public Health. 2023;23(1):1479. 37537530 10.1186/s12889-023-16045-4 PMC10398981

[8] Phelps NH, Singleton RK, Zhou B, Heap RA, Mishra A, Bennett JE, et al. Worldwide trends in underweight and obesity from 1990 to 2022: a pooled analysis of 3663 population-representative studies with 222 million children, adolescents, and adults. Lancet. 2024;403(10431):1027–50. 38432237 10.1016/S0140-6736(23)02750-2 PMC7615769

[9] Victora C, Christian P, Vidaletti L, Gatica-Dominguez G, Menon P, Black R. Revisiting maternal and child undernutrition in low-income and middle-income countries: variable progress towards an unfinished agenda. Lancet. 2021;397(10282):1388–99. 33691094 10.1016/S0140-6736(21)00394-9 PMC7613170

[10] Leroy JL, Frongillo EA. Perspective: what does stunting really mean? A critical review of the evidence. Adv Nutr. 2019;10(2):196–204. 30801614 10.1093/advances/nmy101 PMC6416038

[11] Danaei G, Andrews KG, Sudfeld CR, Fink G, McCoy DC, Peet E, et al. Risk factors for childhood stunting in 137 developing countries: a comparative risk assessment analysis at global, regional, and country levels. PLoS Med. 2016;13(11):e1002164. 27802277 10.1371/journal.pmed.1002164 PMC5089547

[12] Wake S, Baye B, Gondol K. Longitudinal study of growth variation and its determinants in body weight of children aged 1–15 years in Ethiopia. Iran J Pediatr. 2022;32(4):e122662.

[13] Argawu AS, Muniswamy B, Punyavathi B. Analyzing children’s weight growth variations and associated factors in Ethiopia, India, Peru, and Vietnam: using fractional polynomial mixed-effects model. Ethiop J Health Sci. 2024;34(1):27–38. 38957340 10.4314/ejhs.v34i1.4 PMC11217795

[14] Schott WB, Crookston BT, Lundeen EA, Stein AD, Behrman JR. Periods of child growth up to age 8 years in Ethiopia, India, Peru and Vietnam: key distal household and community factors. Soc Sci Med. 2013;97:278–87. 23769211 10.1016/j.socscimed.2013.05.016 PMC3812418

[15] Wilson I, Huttly S, Fenn B. A case study of sample design for longitudinal research: young lives. Int J Soc Res Methodol. 2006;9(5):351–65.

[16] Briones K. A guide to Young Lives rounds 1 to 5 constructed files. Oxford: Young Lives; 2018. https://www.younglives.org.uk. Accessed 2023 Sep 5.

[17] Lives Y. A guide to Young Lives research. Oxford: Young Lives; 2017. https://www.younglives.org.uk. Accessed 2023 Sep 5.

[18] Lives Y. Young Lives survey design and sampling (Round 5): Ethiopia. Oxford: Young Lives; 2018. https://www.younglives.org.uk. Accessed 2023 Sep 5.

[19] Sánchez A, Escobal J. Survey attrition after 15 years of tracking children in four developing countries: the young lives study. Rev Dev Econ. 2020;24(4):1196–216.

[20] Lives Y. Young lives survey design and sampling (round 5): United Andhra Pradesh. Oxford: Young Lives; 2017. https://www.younglives.org.uk. Accessed 2023 Sep 5.

[21] Barnett I, Ariana P, Petrou S, Penny ME, Duc LT, Galab S, et al. Cohort profile: the Young Lives study. Int J Epidemiol. 2013;42(3):701–8. 22617687 10.1093/ije/dys082

[22] Pinheiro J, Bates D. Mixed-effects models in S and S-PLUS. New York: Springer; 2000.

[23] Davidian M. Nonlinear models for repeated measurement data. London: Routledge; 2017.

[24] Pinheiro J, Bates D. Approximations to the log-likelihood function in the nonlinear mixed-effects model. J Comput Graph Stat. 1995;4(1):12–35.

[25] Pinheiro J. nlme: Linear and nonlinear mixed effects models. R package version 3.1–101. 2011. https://cran.r-project.org/package=nlme. Accessed 2023 Sep 5.

[26] Cole TJ, Green PJ. Smoothing reference centile curves: the LMS method and penalized likelihood. Stat Med. 1992;11(10):1305–19. 1518992 10.1002/sim.4780111005

[27] Popkin BM, Corvalan C, Grummer-Strawn LM. Dynamics of the double burden of malnutrition and the changing nutrition reality. Lancet. 2020;395(10217):65–74. 31852602 10.1016/S0140-6736(19)32497-3 PMC7179702

[28] Headey D, Hirvonen K, Hoddinott J. Animal sourced foods and child stunting. Am J Agric Econ. 2018;100(5):1302–19. 33343003 10.1093/ajae/aay053 PMC7734193

[29] Rogol AD, Clark PA, Roemmich JN. Growth and pubertal development in children and adolescents: effects of diet and physical activity. Am J Clin Nutr. 2000;72(2):S521–8. 10.1093/ajcn/72.2.521S 10919954

[30] Tanner JM. Foetus into man: Physical growth from conception to maturity. Cambridge, MA: Harvard University Press; 1990.

[31] Pongou R, Salomon JA, Ezzati M. Health impacts of macroeconomic crises and policies: determinants of variation in childhood malnutrition trends in Cameroon. Int J Epidemiol. 2006;35(3):648–56. 16567342 10.1093/ije/dyl016

[32] Wamani H, Åstrøm AN, Peterson S, Tumwine JK, Tylleskär T. Boys are more stunted than girls in sub-Saharan Africa: a meta-analysis of 16 demographic and health surveys. BMC Pediatr. 2007;7(1):17. 17425787 10.1186/1471-2431-7-17 PMC1865375

