# Supplementary material for: Modeling children’s weight growth trajectories: sex, country, and rural–urban differences in four low- and middle-income countries
Source: BMC Pediatr. 2025 Dec 31;26:100. doi: 10.1186/s12887-025-06459-x (PMC12882441; doi:10.1186/s12887-025-06459-x)
Supplement: Supplementary file 1 — Supplementary material 1. [file 12887_2025_6459_MOESM1_ESM.docx]

**Supplementary Table 1** Young Lives study design, sampling, and data quality procedures (Younger Cohort, 2002–2016)

| Country | Cohort size (Round 1, 2002, Younger cohort) | Sentinel sites (urban/rural) | Recruitment strategy | Follow-up rounds (child age) | Attrition by Round 5 (%) | Data collectors & consent | Anthropometric quality control |
| --- | --- | --- | --- | --- | --- | --- | --- |
| Ethiopia | 2,000 children  (aged 6–18 months) | 20 sentinel sites across Amhara, Oromia, SNNP, and Tigray; a mix of rural villages and urban centers | Semi-purposive selection of poor and diverse communities; random selection of households within clusters | 5 rounds: 2002 (~1y), 2006 (~5y), 2009 (~8y), 2013 (~12y), 2016 (~15y) | **4.5%** | Local survey teams; consent from caregivers, assent from children in later rounds; ethics approvals in Ethiopia and the University of Oxford | Calibrated scales; standardized training; re-measurement of subsamples; ongoing supervision |
| India (Andhra Pradesh & Telangana) | 2,011 children | 20 sites across districts, covering tribal, rural, peri-urban, and urban areas | Purposive sampling of poor communities with diversity; random child selection within sites | Same as above | **3.0%** | Centre for Economic and Social Studies (CESS); caregiver consent; national/state ethics approvals | Standardized protocols; refresher training; 5% subsample re-measured; data entry checks |
| Peru | 2,052 children | 20 sites across coastal, highland, and Amazon regions; rural and urban | Multi-stage sampling, oversampling poor households; random selection within clusters | Same as above | **8.2%** | Grupo de Análisis para el Desarrollo (GRADE); national ethics approval; caregiver consent | Daily calibration of digital scales; duplicate measurements; double data entry |
| Vietnam | 2,000 children | 20 sites in five provinces (urban/rural mix) | Stratified cluster sampling; emphasis on rural poor households; random child selection | Same as above | **2.3%** | Vietnam Academy of Social Sciences; caregiver consent; local ethics approvals | Calibrated equipment; duplicate measurements on subsamples; strict QC checks, retraining |
