# Supplementary material for: Modeling children’s weight growth trajectories: sex, country, and rural–urban differences in four low- and middle-income countries
Source: BMC Pediatr. 2025 Dec 31;26:100. doi: 10.1186/s12887-025-06459-x (PMC12882441; doi:10.1186/s12887-025-06459-x)
Supplement: Supplementary file 2 — Supplementary material 2. [file 12887_2025_6459_MOESM2_ESM.docx]

**Supplementary Table 2** Symbols and parameters in the logistic growth model

| Symbol | Parameter name | Interpretation in this study |
| --- | --- | --- |
| $\boldsymbol{\phi}_{\boldsymbol{1}}$ | Asym (Asymptote) | The estimated maximum average weight (kg) a child approaches in late adolescence. |
| $\boldsymbol{\phi}_{\boldsymbol{2}}$ | Xmid (Inflection point) | The child’s age (years) at which growth rate is fastest; the midpoint of the logistic curve. |
| $\boldsymbol{\phi}_{\boldsymbol{3}}$ | Scal (Scale) | The steepness of the growth curve; smaller values indicate faster changes in weight around the inflection point. |
| $\boldsymbol{f(age)}$ | Logistic growth function | Predicted weight (kg) at a given age. |
