# Supplementary material for: Modeling children’s weight growth trajectories: sex, country, and rural–urban differences in four low- and middle-income countries
Source: BMC Pediatr. 2025 Dec 31;26:100. doi: 10.1186/s12887-025-06459-x (PMC12882441; doi:10.1186/s12887-025-06459-x)
Supplement: Supplementary file 3 — Supplementary material 3. [file 12887_2025_6459_MOESM3_ESM.docx]

**Supplementary Table 3** Sensitivity analyses. Comparison of main results with models excluding outliers and alternative specifications.

| Model specification | Asym (kg) | Xmid (years) | Scal | Interpretation |
| --- | --- | --- | --- | --- |
| Main model (3-parameter logistic,  log-transformed, with random effects on all parameters) | 87.4 | 13.9 | 1.8 | Reference |
| Excluding statistical outliers (n ≈ 200 dropped) | 87.0 | 13.8 | 1.8 | Nearly identical estimates |
| Without random effect on Xmid | 86.9 | 14.0 | 1.9 | Slight change, fit worse (higher AIC) |
| Alternative family: Gompertz | 125+ | — | — | Implausible asymptote (discarded) |
